# Fresh and Frozen Tissue-Engineered Three-Dimensional Bone–Ligament–Bone Constructs for Sheep Anterior Cruciate Ligament Repair Following a 2-Year Implantation

**DOI:** 10.1089/biores.2016.0032

**Published:** 2016-10-01

**Authors:** Vasudevan Mahalingam, Edward M. Wojtys, Deneen M. Wellik, Ellen M. Arruda, Lisa M. Larkin

**Affiliations:** ^1^ Department of Molecular and Integrative Physiology, University of Michigan, Ann Arbor, Michigan.; ^2^ Medsport Sports Medicine Program, Department of Orthopaedic Surgery, University of Michigan, Ann Arbor, Michigan.; ^3^ Department of Internal Medicine, Division of Molecular Medicine and Genetics, University of Michigan, Ann Arbor, Michigan.; ^4^ Department of Mechanical Engineering, University of Michigan, Ann Arbor, Michigan.; ^5^ Department of Biomedical Engineering, University of Michigan, Ann Arbor, Michigan.; ^6^ Program in Macromolecular Science and Engineering, University of Michigan, Ann Arbor, Michigan.

**Keywords:** anterior cruciate ligament reconstruction, scaffold-free, tissue engineering

## Abstract

Injuries to the anterior cruciate ligament (ACL) often require surgical reconstruction utilizing tendon grafts to restore knee function and stability. Some current graft options for ACL repair are associated with poor long-term outcomes. Our laboratory has fabricated tissue-engineered bone–ligament–bone (BLB) constructs that demonstrate native ligament regeneration and advancement toward native ACL mechanical properties in a sheep ACL reconstruction model. Prior work has shown that freezing BLBs as a method of preservation resulted in similar outcomes compared with fresh BLBs after 6-month implantation. The purpose of this study was to evaluate the long-term efficacy of fresh and frozen BLBs. We hypothesized that both fresh and frozen BLBs would show continued regeneration of structural and functional properties toward those of native ACL after a 2-year implantation. Following removal of the native ACL, fresh (*n* = 2) and frozen (*n* = 2) BLBs were implanted arthroscopically. After 2 years of recovery, sheep were euthanized and both the experimental and contralateral hindlimbs were removed and radiographs were performed. Explanted knees were initially evaluated for joint laxity and were then further dissected for uniaxial tensile testing of the isolated ACL or BLB. Following mechanical testing, explanted contralateral ACL (C-ACL) and BLBs were harvested for histology. Two years post-ACL reconstruction, fresh and frozen BLBs exhibited similar morphological and biomechanical properties as well as more advanced regeneration compared with our 6-month recovery study. These data indicate that an additional 1.5-year regeneration period allows the BLB to continue ligament regeneration *in vivo*. In addition, freezing the BLBs is a viable option for the preservation of the graft after fabrication.

## Introduction

Anterior cruciate ligament (ACL) injuries are a major healthcare burden. Unfortunately, the highest incidence and rate of increase are found in teenagers and young adults.^1^ This is particularly worrisome considering that ∼50% of patients develop osteoarthritis within 20 years after ACL injury,^2^ resulting in debilitating conditions over a longer period of time and diminished quality of life. Current repair strategies utilize replacement tendon grafts that are not mechanically equivalent to the native ACL and have limited ability to biointegrate with the host. These grafts, while allowing for initial stability and rehabilitation, are generally too stiff, can cause tunnel widening, and exorbitant forces on the knees resulting in potential rerupture or other damage,^3^ and may not allow for endogenous tissue regeneration leading to attenuation of the graft and degeneration of the knee over time.^4^

Our laboratory has developed tissue-engineered bone–ligament–bone (BLB) constructs derived from bone marrow stromal cells. Previous work with these constructs as grafts for ACL reconstruction in a large animal (sheep) model have shown clear improvements in mechanical properties and biointegration over bone–patellar tendon–bone grafts after 6 months implantation.^5^ Furthermore, two additional 6-month implantation studies have compared allogeneic and autologous cell-derived BLB constructs^6^ as well as fresh and frozen BLB constructs.^7^ These studies showed minimal differences between allogeneic and autogenic, fresh and frozen grafts, permitting more versatility in the use of the constructs as ACL grafts. Recent work investigating the regenerative process of the constructs in acute implantation studies showed that dramatic cellular turnover occurs within days of implantation, and by 28 days, all cells in the explanted grafts are host derived. The ability of BLB constructs to stimulate early integration of endogenous cells may contribute to their enhanced recovery of native ACL phenotypes over other graft technologies.

It was not known, however, whether these improvements in the short term would translate to better outcomes in the long term, particularly with the maintenance of knee stability and prevention of post-traumatic knee degeneration. Furthermore, the viability and fate of previously frozen BLBs at extended time points was also unknown. Therefore, the purpose of this study was to examine the growth and regeneration of fresh and frozen BLB constructs after a 2-year implantation period. We hypothesized that both fresh and frozen BLB grafts would show continued regeneration of structural and functional properties of the graft tissue toward native ACL properties, and similarly to our previous 6-month study, fresh and frozen BLB grafts would show no differences in the ability to regenerate and maintain native ACL function.

## Materials and Methods

### Animal use and experimental design

All animal care and animal surgery procedures were performed in accordance with *The Guide for Care and Use of Laboratory Animals*: *Eighth Edition*^8^; the experimental protocol was approved by the University Committee for the Use and Care of Animals at the University of Michigan. Animals were acclimated to the Unit for Laboratory Animal Medicine (ULAM) husbandry facilities at the University of Michigan for at least 1 week before any procedure and were given access to food and water *ad libitum*. Bone marrow stromal cells (BMSCs) were harvested from iliac crest marrow aspirations on adult female Black Suffolk sheep to fabricate our BLB tissue-engineered constructs for use as grafts as in our previously described model for sheep ACL reconstruction.^5–7^ Two sheep were implanted with fresh BLBs and another two were implanted with frozen BLBs. Recovery was allowed for 2 years.

### Preparation of cell culture supplies

The media and reagents used in this experiment have all been previously described.^5^ All solutions and media were prepared and stored at 4°C and were warmed to 37°C in a heated bead bath before use. Briefly, growth medium (GM) consisted of 78% Dulbecco's modified Eagle's medium (DMEM; Gibco, Grand Island, NY), with 20% fetal bovine serum (FBS; Gibco), 2% antibiotic–antimycotic (ABAM; Invitrogen, Grand Island, NY), 6 ng/mL basic fibroblast growth factor (bFGF; PeproTech, Rocky Hill, NJ), 0.13 mg/mL ascorbic acid-2-phosphotase (Sigma-Aldrich, St. Louis, MO), and 0.05 mg/mL l-proline (Sigma-Aldrich); differentiation medium (DM) consisted of 91% DMEM, 7% horse serum albumin (HS; Gibco), 2% ABAM, 0.13 mg/mL asc-2-phos, 0.05 mg/mL l-proline, and 2 ng/mL transforming growth factor-beta (TGF-β; PeproTech). For the culture of bone-like constructs, 10^−8^ M dexamethasone (DEX; Sigma-Aldrich) was added to GM and DM.^9^

Construct dishes were prepared as described previously^5^ to house and constrain the formed three-dimensional constructs. Briefly, 100-mm diameter cell culture plates were filled with 12 mL Sylgard (Dow Chemical Corp., Midland, MI; type 184 silicon elastomer) and allowed to cure for 3 weeks at room temperature. Before use, plates were decontaminated with UV light (wavelength 253.7 nm) for 60 min and rinsed with 70% EtOH and DPBS.

### Isolation and expansion of BMSCs

Bone marrow aspirates were collected from the iliac crest of a sheep using a Monoject Illinois aspiration needle (Sherwood Medical Company, St. Louis, MO) with the animal under general anesthesia. The collected aspirate was filtered through a 100 mm filter to remove debris and combined into a total volume of 15 mL with an equivalent volume of DPBS added. A layer of 15 mL Ficoll-Paque Premium (MNC; GE Healthcare, Munich, Germany) was carefully layered on top of the aspirate and the solution was centrifuged (AccuSpin FR; Beckman Coulter, Inc., Fullerton, CA) at room temperature at 600 *g* for 30 min to separate the aspirate components by density. The upper layer of plasma was removed and the mesenchymal cells contained in the middle mononuclear cell layer were transferred into a new conical filled with DPBS. The remaining aspirate contents of the conical were discarded. The purified isolate was then centrifuged at 500 *g* for 10 min and the supernatant removed. An equivalent volume to the pellet of ACK Lysing Buffer (Gibco) was added and mixed for 30 sec to lyse any remaining red blood cells. The conical was then filled with DPBS and centrifuged at 400 *g* for 5 min to wash the cells. After the supernatant was removed, the pellet was resuspended in 20 mL GM and a cell count was taken. BMSCs were then plated at 40,000 cells/cm^2^ in cell culture dishes.

### Fabrication of BLBs

The BMSCs were expanded into ligament and bone lineages.^5,9,10^ Passage-3 cells in the ligament pathway and passage-4 cells in the bone pathway were seeded at a density of 21,000 cells/cm^2^ and switched to DM after 8 days plating. After 2 days in DM, the bone monolayers were rolled with sterile tweezers into a cylinder shape and transferred to Sylgard plates. After one to two additional days, the bone constructs were ready to be incorporated into a ligament monolayer to create our BLB. Each confluent ligament monolayer was removed intact from the cell culture plate surface and transferred to Sylgard plates and pinned back into a single layer. Bone constructs were pinned on each end of the ligament monolayer and were subsequently wrapped with the ligament monolayer. The length of the BLB adjusted with minutien pins to the desired length of ∼60 mm comprised of at least a 30 mm ligament portion and two 15 mm bone ends. Four of these constructs were placed side-by-side and allowed to fuse. The DM was changed every 2–3 days. After 2 weeks of formation, two fused sets of four constructs were combined for an implantation width of ∼5 mm at the ligament region. After an additional 1 week in culture, the fully formed BLB was ready for implantation. All tissues were constrained using minutien pins at set distances at all times during 3-D culture. Before implanting, the bone ends of the BLB were threaded with nonabsorbable 5-0 silk suture to allow for passage into the bone tunnel and fixation onto the periosteum.

Fully formed BLBs were placed into individual Sylgard dishes and stored in a portable incubator for transport into surgical suites. Fresh BLBs were used directly from the incubator. Frozen BLBs were prepared by first sealing the lid of the Sylgard dish with paraffin film. The entire dish was then placed into an −80°C freezer to ensure that the construct was completely frozen for a minimum of 1 h. Before implantation, plates were retrieved from the freezer and allowed to thaw in a 37°C bead bath. After complete thawing of the media, paraffin film was removed and plates were stored in a cell culture incubator at 37°C until implantation.

### Surgical procedure

ACL reconstructions were performed arthroscopically at the ULAM Animal Surgery Operating Rooms at the University of Michigan as described previously.^6,5,11^ Briefly, after induction of general anesthesia and preparation of the surgical site, the native ACL of the left limb was removed with remnants of the stump on both the femur and tibia used to aid in anatomical positioning of the BLB. Drill guides were used to position Steinmann pins for precise anatomic placement at the center of the tibial and femoral footprints. Bone tunnels (5–6 mm) were drilled using cannulated reamers over the pins. The BLB was then passed through the bone tunnels using suture until the threaded bone ends of the BLB were not visible arthroscopically in the intra-articular space. The proximal and distal ends were then secured with suture to the periosteum. Incisions were closed with staples and the entire surgical site was sprayed with AluShield (Neogen Corp., Lansing, MI). The contralateral (right) limb served as a control. Animals were monitored daily at the ULAM facility for 2 weeks, after which staples were removed and the animals were sent to a large outdoor pen to allow greater mobility.

### Explantation

After 2 years implantation, sheep were returned to the ULAM facility. Sheep were euthanized and both hindlimbs were harvested with the knee joints intact. The intra-articular space was opened by cutting the patellar tendon and reflecting the patella to collect synovial fluid for analysis. Radiographic examinations were then taken in the anteroposterior and lateral views. Anterior drawer testing of the knee was then performed with the knee capsule intact. The knee was subsequently dissected to the BLB or contralateral ACL (C-ACL) for uniaxial tensile testing. Following anterior drawer and uniaxial tensile testing, the BLB and C-ACL were resected from their tibial and femoral bone insertions and harvested for histology. Detailed methods of these procedures follow.

### Knee laxity testing

Knee laxity was measured using a custom-designed anterior drawer tester.^12^ The bones were potted in grips using a polymer that became malleable when heated and hardened to its conformed shape when allowed to cool. The bone and hardened polymer were secured with two ¼″-20 screws in the grips and mounted onto an MTS 810 servohydraulic test system with a 25 kN load cell. Ink markings were placed at a known distance onto the femur and tibia grips for displacement tracking. The test comprised of a 0.5 mm/s extension until a 50 N force was achieved. Images were collected with a Grasshopper IEEE-1394b digital camera (Point Grey, Richmond, British Columbia, CA) and analysis for displacement was determined using MetaMorph software.

### Uniaxial tensile testing

Knee tissue was further dissected away leaving only the BLB or C-ACL attached at both tibial and femoral insertions. The length of the ligament as well as the width and thickness of the proximal, middle, and distal regions were measured and recorded. The cross-sectional areas from these three locations were averaged and used as the representative area for stress calculations. The knee was repositioned for a flexion angle of 30° by fixing the tibia and femur grips at 90° and 60°, respectively in the sagittal plane to put the ligament in a uniaxial loading configuration. Graphite powder was blown onto the specimen to create a surface pattern for optical displacement measurement using digital image correlation (DIC) to compute full-field strain contours. Uniaxial tension tests at a strain rate of 0.05/s for a loading time of 7.5 sec were then conducted on the BLB and ACL specimens to obtain the stiffness using previously developed testing protocols.^5^ A Photron high-speed camera was used for synchronized force and image acquisition with a custom-developed LabVIEW program. The load–unload cycle for each specimen was run in triplicate. VIC-2D Software (Correlated Solutions, Columbia, SC) was used for DIC analysis.

### Histological analysis of explanted BLB and C-ACL

For histological preparation, samples were fixed in 4% paraformaldehyde for 48 h and frozen in TBS™ TFM™ Colored Tissue Freezing Medium at −80°C until processing. Each tissue sample was sectioned at 12 μm using a cryostat, placed onto Superfrost Plus microscopy slides, and stored at −20°C. Longitudinal sections were taken from the mid-substance of the explanted grafts and contralateral ACL. For morphological characteristics, sections were stained with Hematoxylin and Eosin (H&E). Picrosirius Red staining (Polysciences, Inc., Warrington, PA) was used for analysis of collagen content and fiber organization. Semiquantitative analysis was performed of the collagen fiber birefringence using previously established methods.^11^ Under uniform conditions, three serial sections stained for Picrosirius Red were imaged at 10× magnification in a single session under monochromatic polarized light rotated in the plane for maximum brightness. Images were imported using ImageJ software into 8-bit gray scale showing collagenous material representing a gray scale value 1–255 and noncollagenous components as dark with a gray scale value of 0. Mean gray scale values of nine randomly selected rectangular areas (50 × 50 μm) from each section of each animal were obtained using ImageJ to assign a brightness value for comparison.

### Statistical analysis

Comparisons among the three groups for mechanical properties and collagen birefringence were done using one-way analysis of variance with Tukey's *post hoc* test using GraphPad Prism 6 software. A *p-*value <0.05 for all statistical tests was considered significant. All data were reported as mean ± standard deviation.

## Results

### Explantation

Before euthanasia, the sheep did not appear to have any issues with gait as assessed by the veterinary staff at the University of Michigan. Examination of the knee joints did not show any deformity or abnormalities. Blood and synovial fluid analysis did not indicate signs of infection.

### Radiograph examination

**FIG. 1.**
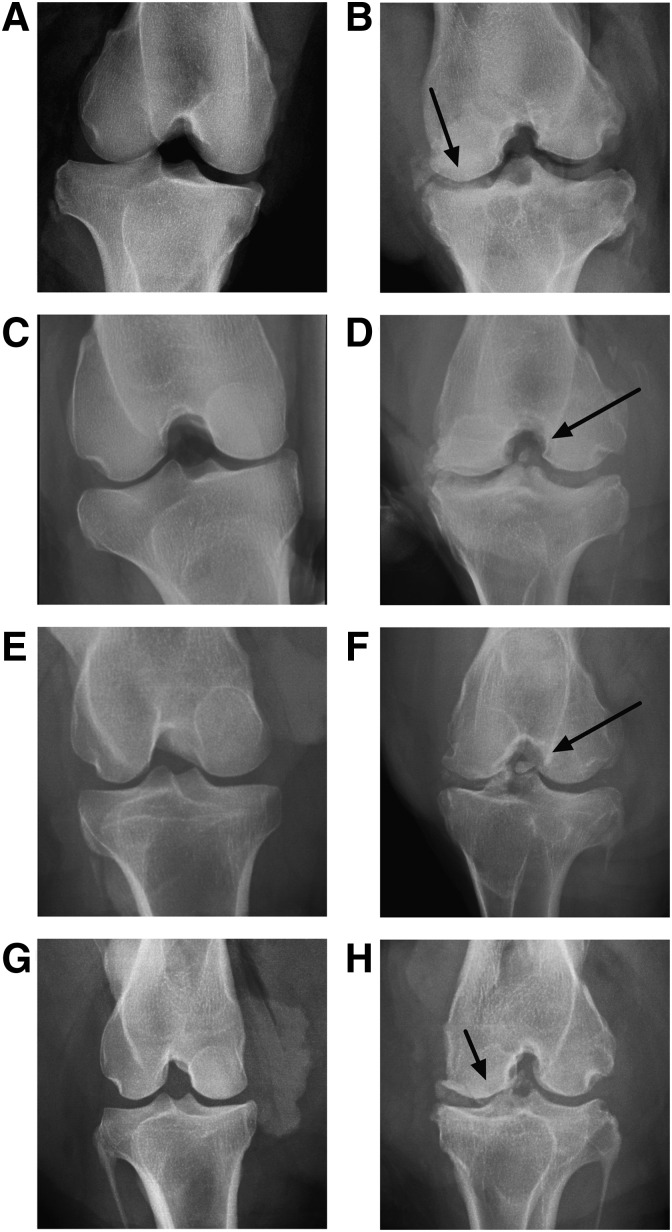
Explant radiographs. Anteroposterior views of contralateral and graft knees from BLB graft explants after 2-year implantation using fresh **(B, D)** and frozen **(F, H)** BLB constructs and associated contralateral knee **(A, C, E, G)**. Arrows indicate loose bone fragments and osteophyte formations. BLB, bone–ligament–bone.

Radiographs of the explanted graft knees showed osteophyte formations and loose bone fragments present in the knees (Fig. 1B, D, F, H). The contralateral knee radiographs did not have these characteristics (Fig. 1A, C, E, G).

### Gross morphology and structure

**FIG. 2.**
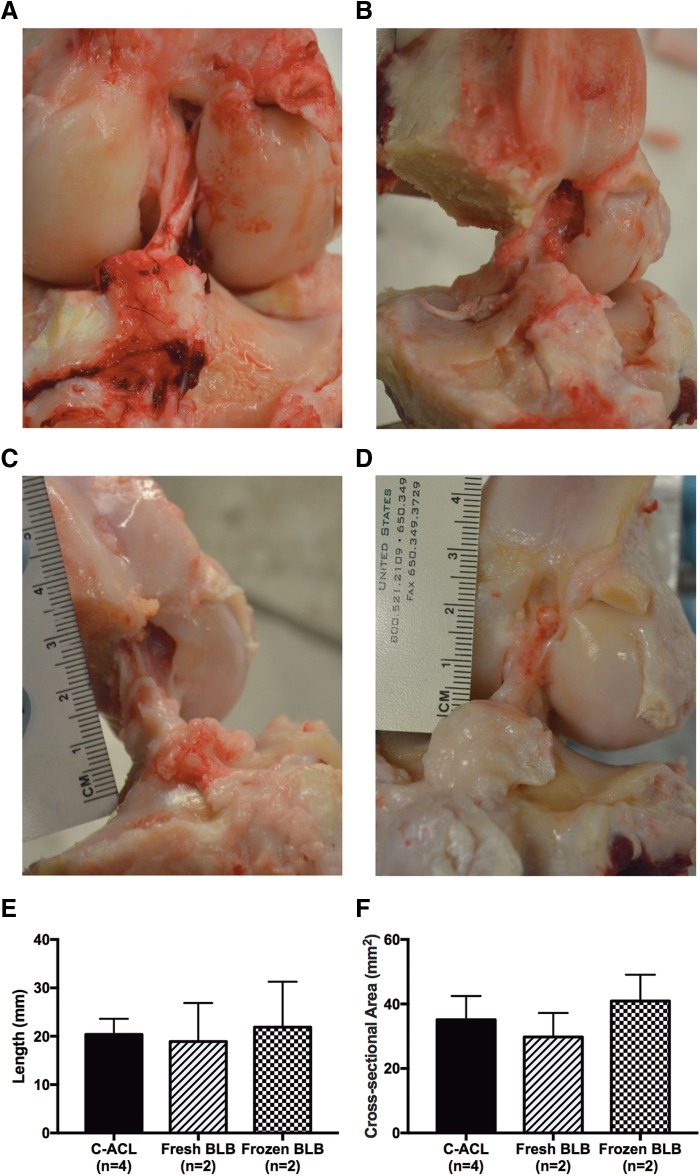
Structure and morphology of explanted BLB grafts. Fresh **(A, B)** and frozen BLB **(C, D)** knees had tissue spanning the intra-articular space with attachments at appropriate tibial and femoral insertion sites. Length **(E)** and cross-sectional area **(F)** were not different from the contralateral ACL (C-ACL). ACL, anterior cruciate ligament.

Upon dissection of the knee to isolate the BLB graft, both fresh (Fig. 2A, B) and frozen (Fig. 2C, D) repaired knees showed robust ligament tissue spanning the intra-articular space at the appropriate femoral and tibial insertions. Gross inspection of knee menisci did not indicate any tearing or injury. The average length of the fresh BLB grafts (18.9 ± 8.0 mm) and frozen BLB grafts (21.9 ± 9.4 mm) were not significantly different than the C-ACL (20.3 ± 3.2 mm) (Fig. 2E). Similarly, the average cross-sectional area of fresh and frozen BLB grafts, 29.8 ± 7.5 and 40.9 ± 8.2 mm^2^, respectively, were not significantly different than the C-ACL (35.1 ± 7.4 mm^2^) (Fig. 2F).

### Knee laxity

**FIG. 3.**
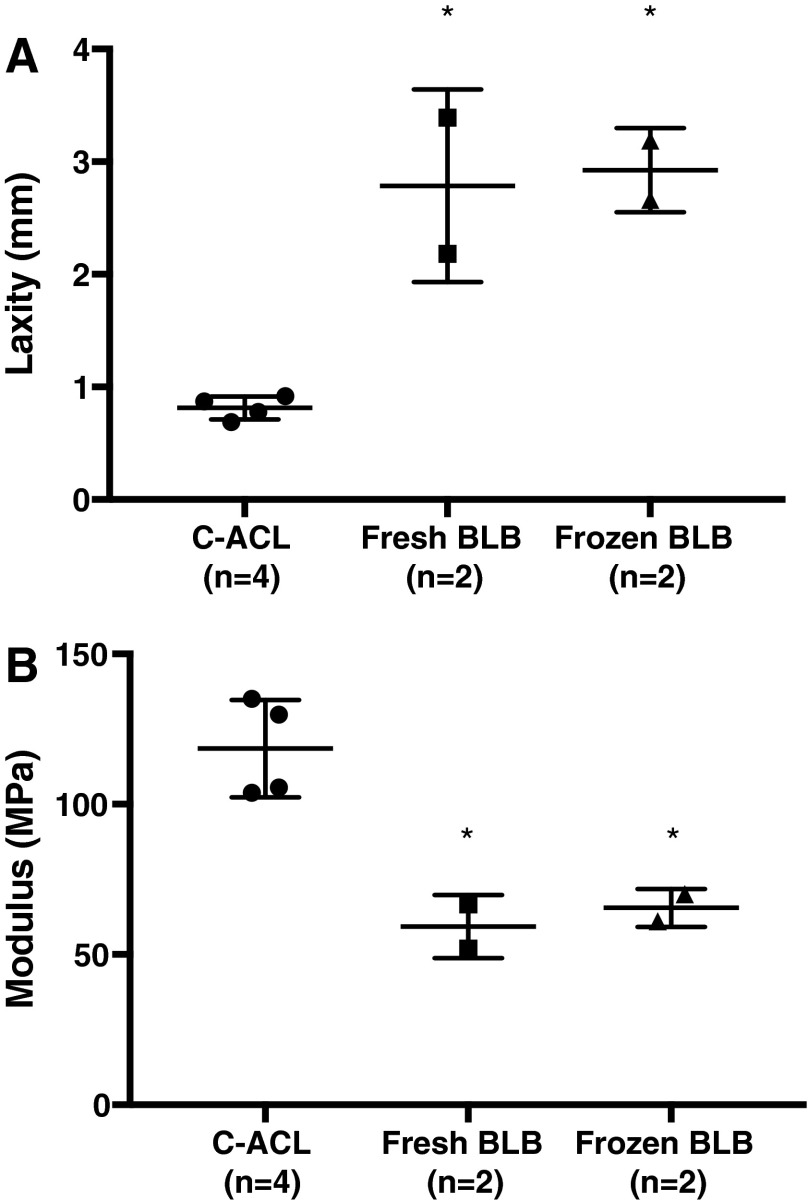
Laxity and modulus measurements of explanted fresh and frozen BLB grafts. Graft laxities were significantly higher than C-ACL **(A)**. The tangent moduli **(B)** of the grafts were significantly lower than the C-ACL, but taken together reached 53% of the C-ACL. Fresh and frozen BLB grafts were not different for either test. *Significantly different from C-ACL (*p* > 0.05).

The laxity of knees implanted with fresh BLB grafts were 2.2 and 3.4 mm, whereas that of the frozen BLB graft knees were 2.7 and 3.2 mm. The C-ACL knee laxity averaged 0.8 ± 0.1 mm (*n* = 4) (Fig. 3A). There was no significant difference between fresh and frozen knee laxity, but both groups had significantly increased laxity compared with the C-ACL knees.

### Modulus analysis

The tangent moduli (slope of the stress–strain curve at a specified strain range) taken at the linear portion of the stress–strain curve of the explanted fresh BLB grafts were 67 and 52 MPa, and the tangent moduli of the frozen BLB grafts were 70 and 61 MPa (*n* = 2) at a strain range of 0.04–0.10 (Fig. 3B). The tangent modulus data indicated no significant differences between the two graft types (*p* > 0.05). Combined, the grafts recovered 53% of the average C-ACL modulus, which was 118 ± 16 MPa (*n* = 4) at a strain range of 0.04–0.10.

### Histological analysis

**FIG. 4.**
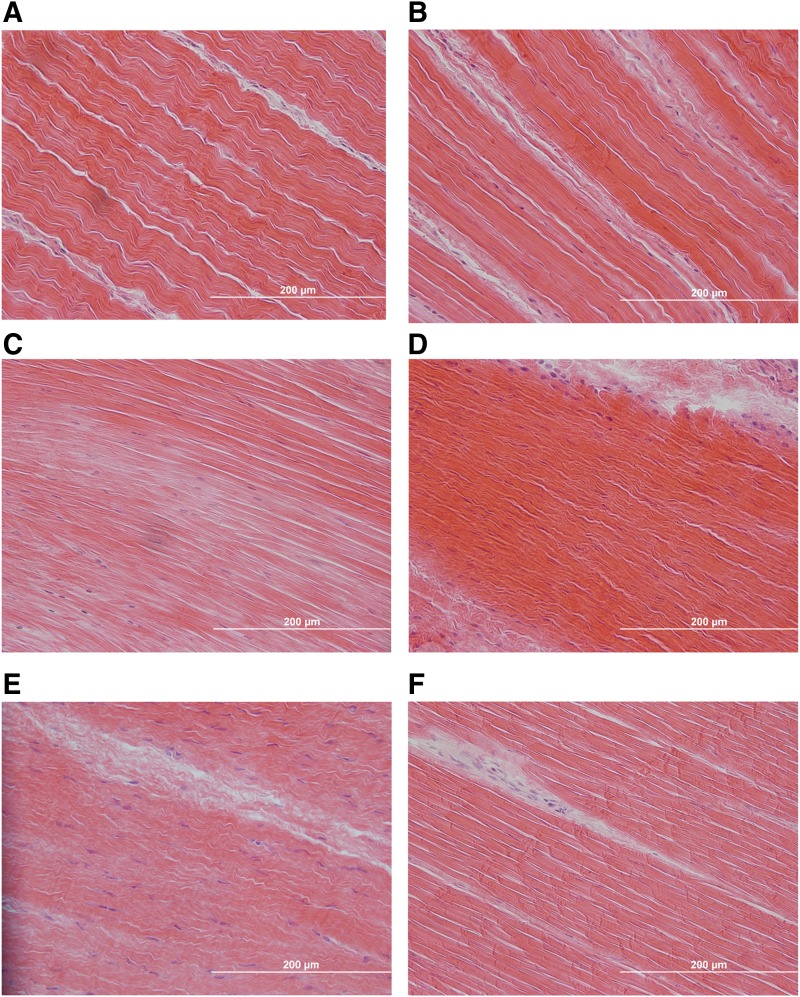
H&E staining of C-ACL **(A, B)** and explanted fresh **(C, D)** and frozen **(E, F)** BLB graft. Grafts showed advanced remodeling and tissue regeneration exhibited by a native-like collagen crimp pattern with fibers aligned along the longitudinal axis of the tissue. 40× magnification. Scale bar = 200 μm. H&E, Hematoxylin and Eosin.

**FIG. 5.**
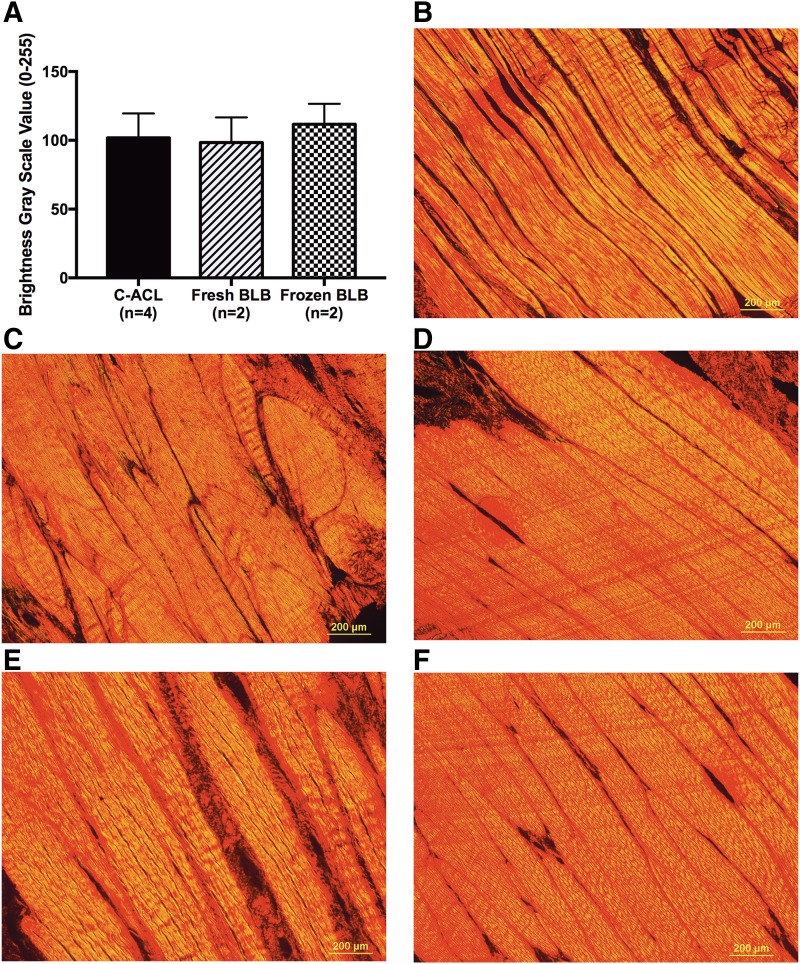
Collagen organization of C-ACL and explanted graft-repaired tissues. Mean gray scale values were not significantly different between tissue types **(A)**. Representative images of Picrosirius Red staining used for semiquantitative analysis of collagen birefringence from fresh **(C, D)** and frozen **(E, F)** BLB graft repaired tissues compared with C-ACL **(B)**. 10× magnification Scale bar = 200 μm.

H&E staining of the fresh and frozen BLB grafts showed a native-like crimp pattern and a ligament tissue structure with collagen fibers aligned along the longitudinal axis of the tissue (Fig. 4). Semiquantitative analysis of collagen birefringence from Picrosirius Red-stained sections revealed no significant difference in mean gray scale values among C-ACL, fresh and frozen BLB tissues (Fig. 5).

## Discussion

In this study, we showed that after a long-term 2-year recovery period, the BLB grafts showed improvement in the regeneration of the repaired ACL compared with our previously reported 6-month recovery study. Additionally, similar to our 6-month study, we once again showed that the outcomes of fresh compared with frozen grafts had indistinguishable mechanical and histological results. Thus, for the purposes of increasing our numbers in this study for statistical comparisons to our previous 6-month study, we have combined the results of the fresh and frozen grafts in the subsequent analysis and discussion. Data from this 2-year study confirm our previous 6-month work showing that our tissue-engineered BLB constructs have favorable outcomes as replacement grafts for ACL reconstruction in a sheep model and that an additional 18-month period for recovery led to increased tissue regeneration and enhanced mechanical properties of the regenerated ligament tissue.

The histological evaluation of the regeneration in the repaired ligaments in this study showed native collagen fiber alignment, cellularity, and vascularity comparable to the contralateral control ligaments. This indicated significant enhancement in tissue regeneration compared with the ligament in our 6-month study that showed significantly lower collagen alignment in the repaired versus contralateral control tissue. These data show that the BLB grafts continue to undergo remodeling and advancement in phenotype toward native ACL between 6 months and 2 years, and that by 2 years, the regeneration has reached near native morphology. Importantly, the BLBs in the 6-month study had already reached the length, and the cross-sectional area (both at the mid-substance and enthesis) of the native contralateral ACL and the BLB grafts in this study did not continue to increase in size with more time *in vivo*.

The modulus data show that the ACLs repaired with our BLB in this 2-year study regenerated 55% of the stiffness of the contralateral control ACL. This is a significant increase in ligament regeneration compared with that observed in the 6-month study, where we only observed a 25% restoration of contralateral ACL modulus. This increase in modulus was concurrent with an advancement in the phenotype of the regenerated ligament that was nearly indistinguishable from the native contralateral ligament suggesting that by the 2-year recovery time point, the repaired ligament had reached its maximum regenerative potential and that further advancement of the mechanical properties of an injured ACL with our graft may need an intervention before the 2-year period. In addition, the average knee laxity in the 2-year and the 6-month repairs were not significantly different. Current studies suggest that a postoperative rehabilitation program, with the appropriate balance of activity, can be the difference between a repair's success or failure,^13^ and may be worth implementing in future studies to further advance mechanical properties of the repair tissue.

The loose bone fragments and osteophyte formations observed in the radiographs of the BLB graft knees indicated degenerative changes in the repaired knees. These signs of degeneration were not observed in the contralateral knees, suggesting that the changes were associated with the surgical procedure or the lack of appropriate knee mechanics during the recovery of the repaired knee. It is possible that the degeneration may have occurred due to the surgical procedure and not due to the lack of mechanical integrity on the repair side. A potential cause is the drilling involved during the surgical procedure, which may have led to loose bone fragments. A previous study in sheep demonstrated the effect of kinematic changes on osteoarthritis development following ACL repair. The sham control group in this study underwent an identical surgical procedure, but without detachment of ACL, showing osteophyte formations after 20 weeks of recovery similar to the experimental repair group.^14^ Future work will need to be done to delineate the degenerative changes associated with the surgical procedure from the graft repair, and whether these changes are alleviated with implementation of postoperative rehabilitation programs.

The results of this study add to previous work highlighting the efficacy of our BLB grafts in ACL repair by confirming the graft's viability and continued regeneration over a longer, 2-year recovery period. We also showed that fresh and frozen BLB constructs have similar outcomes allowing for simple deep freezing as an acceptable method of tissue preservation.

## Abbreviations Used

ACL: anterior cruciate ligament
BLB: bone–ligament–bone
C-ACL: contralateral ACL
DIC: digital image correlation
DM: differentiation medium
GM: growth medium
H&E: Hematoxylin and Eosin
ULAM: Unit for Laboratory Animal Medicine
